# The Usefulness of Extemporaneous Cytological Examination in Imaging-Guided Biopsies

**DOI:** 10.3390/ani15233489

**Published:** 2025-12-03

**Authors:** Andrea Rubini, Francesca Del Signore, Massimo Vignoli, Arianna Miglio, Martina Rosto, Andrea De Bonis, Rossella Terragni, Domenico Santori, Morena Di Tommaso

**Affiliations:** 1 Department of Veterinary Medicine, University of Teramo, Piano d’Accio, 64100 Teramo, Italy; fdelsignore@unite.it (F.D.S.); mrosto@unite.it (M.R.); adebonis@unite.it (A.D.B.); dsantori@unite.it (D.S.); mditommaso@unite.it (M.D.T.); 2 Department of Veterinary Medicine, University of Perugia, Via San Costanzo 4, 06124 Perugia, Italy; arianna.miglio@unipg.it; 3 Pet Care Veterinary Clinic, 40133 Bologna, Italy; terragni.rossella@gmail.com

**Keywords:** extemporaneous cytology, imaging-guided biopsy, ultrasound, computed tomography

## Abstract

This study investigates the usefulness of extemporaneous cytological examination performed during imaging-guided biopsies in dogs and cats. In veterinary diagnostics, imaging techniques such as ultrasound and computed tomography are essential for identifying lesions, but they cannot distinguish between inflammatory and neoplastic processes. Biopsies are therefore required, and this study explores whether immediate cytological evaluation of tissue cores, rolled onto slides and examined under a microscope, can help determine sample adequacy and reduce the number of biopsies needed. Seventy-nine animals with soft tissue or bone masses underwent ultrasound or CT-guided biopsies, followed by extemporaneous cytology and histopathological analysis. Cellular material representative of the lesion was identified in 81.1% of cases, allowing clinicians to conclude the procedure without further sampling. The diagnostic accuracy of cytology compared to histology was 68.3%, with particularly high accuracy for lipomas, melanomas, and mast cell tumors. When excluding non-diagnostic samples due to poor preparation or lesion characteristics, the accuracy reaches 92.1%. These findings suggest that extemporaneous cytology is a useful tool for verifying sample adequacy in real time, potentially reducing procedural risks and improving diagnostic efficiency. While histology remains the gold standard, this method offers immediate feedback and may enhance clinical decision-making in veterinary interventional diagnostics.

## Simple Summary

Diagnosing masses in dogs and cats often requires biopsies guided by imaging techniques such as ultrasound or computed tomography. This study evaluates the usefulness of extemporaneous cytology, a rapid method where tissue samples collected during biopsies are immediately rolled onto slides and examined under a microscope. The aim is to quickly verify whether the sample contains enough cellular material to represent the lesion, helping clinicians decide whether to continue or stop the biopsy procedure. Seventy-nine animals were enrolled, and in 81% of cases, the cytological slides showed adequate cellularity, allowing the procedure to be safely concluded. This approach is especially valuable when biopsies are performed in risky anatomical areas, as it can reduce the number of samples needed and lower the risk of complications. Additionally, the diagnostic accuracy of this method was 68%, with particularly high accuracy for lipomas, melanomas, and mast cell tumors. Although histology remains the gold standard for definitive diagnosis, extemporaneous cytology offers immediate feedback and can support clinical decision-making. With careful sample preparation, this technique may improve diagnostic efficiency and animal safety in veterinary practice.

## 1. Introduction

Diagnostic imaging enables the detection of masses or lesions affecting the soft and/or hard tissues in dogs and cats. However, it does not allow us to differentiate inflammatory/infectious conditions from neoplastic processes. Therefore, cytological or histopathological examinations are required to achieve an accurate diagnosis, establish a prognosis, and plan appropriate treatment [1].

In this context, image-guided interventional procedures play a crucial role in obtaining a definitive diagnosis. To date, several interventional procedures are well known and routinely used in veterinary medicine for dogs and cats to collect samples for cytological or histopathological examinations under imaging guidance. Sample collection can be performed under ultrasound (US), computed tomography (CT), fluoroscopy, or magnetic resonance imaging (MRI) guidance. US and CT are the modalities of choice in interventional diagnostics [2].

Among the interventional diagnostic procedures in dogs and cats, we can mention fine-needle aspiration (FNA) or fine-needle biopsy (FNB) and tru-cut biopsy (TCB) performed under ultrasound or computed tomography guidance. These are routine procedures in small animal medicine that allow for precise needle placement [3,4]. It is worth noting that there are important differences between the performance and interpretation of biopsies obtained with fine needles (≥20 G) and those with larger-core needles (≤18 G) [2]. For example, as reported by Giordano and colleagues [5], liver and kidney biopsies can detect feline infectious peritonitis lesions in cats, but false negatives and poor samples are fairly common, especially in immunochemical tests. Examining both TCB and FNA samples from the same organ can improve sensitivity. In the case of FNA, cytology allows us to read the slide quickly and without the need to sedate the patient, but at the expense of diagnostic accuracy [6,7]. On the other hand, tru-cut biopsy (in the case of soft tissue) or bone needle biopsy (in the case of bone tissue) proves to be a much more reliable method given the greater amount of tissue collected, which allows for histological study of the sample; however, it is a more invasive test and requires slightly longer time to obtain results. The diagnostic accuracy differs considerably between the two methods, FNA and TCB, respectively, at 67.7% and 95.2%, as demonstrated by a recent study conducted on 62 patients with thoracic masses [7]; 65% and 83%, respectively, as demonstrated by a study conducted on 30 intrathoracic injuries in dogs and cats [6]. In veterinary medicine, there are several studies that examine the diagnostic accuracy of cytology using FNA and imaging-guided tru-cut biopsy of masses and nodules in dogs and cats [6,7,8]. In one study, for example, the concordance between cytology and histology in the diagnosis of metastatic lymph nodes was evaluated. The authors of this study concluded that lymph node cytology in dogs and cats is fairly specific but not very sensitive, with false negatives occurring mainly in certain tumors such as T-cell lymphomas, sarcomas and mast cell tumors [9].

However, to the author’s knowledge, there are currently no studies in veterinary medicine that have investigated the usefulness of extemporaneous cytology by rolling the tissue core at the time of imaging-guided biopsies.

The originality of this project lies in the fact that this procedure could bring benefits to clinical practice by limiting the number of biopsies to be performed and thus limiting the risks associated with them.

The aim of the following study is therefore twofold. Firstly, it aims to demonstrate the usefulness of extemporaneous cytology when performing imaging-guided biopsies, from the same biopsy material collected, as the presence of cellularity in cytology allows us to terminate biopsy procedures, which is particularly important in dangerous locations, while the lack of cellularity or the presence of necrotic material should suggest that further biopsies be taken.

Another important objective is to evaluate the diagnostic accuracy of cytology obtained from tissue core rolling, and the correspondence between the cytological diagnosis (when possible) and the histopathological diagnosis.

## 2. Materials and Methods

This prospective study was conducted on dogs and cats brought to the diagnostic units of the University Veterinary Teaching Hospital (OVUD) of the Department of Veterinary Medicine in Teramo and the Pet Care Veterinary Clinic in Bologna, from May 2021 to May 2022.

All procedures were performed in accordance with national animal welfare legislation (Legislative Decree No. 26, 3 March 2014) and the study was approved by the Ethics Committee of the Department of Veterinary Medicine of the University of Teramo, protocol No. 803 of 14 January 2022.

Dogs and cats referred for diagnostic imaging procedures requiring a biopsy due to the presence of a soft tissue or bone mass were prospectively enrolled. The data recorded included identification (breed, age and sex), location of the lesion, cytological description of the slides, diagnosis of extemporaneous cytology and final histological diagnosis. All patients were examined by the referring physician and then taken to the two above-mentioned facilities for diagnostic evaluation of the lesions. Image-guided biopsies were performed after sedation of each animal, and all procedures were performed on an outpatient basis. Prior to sedation, each animal underwent a pre-anesthetic evaluation by a board-certified anesthetist, including a cardiopulmonary physical examination, hematologic and biochemical testing, and a coagulation profile with platelet count; all values were within normal limits.

For ultrasound-guided biopsies, the animals underwent different premedication protocols. In dogs [0.2 mg/kg methadone (Semfortan, Dechra Pharmaceuticals PLC, Northwich, UK), 5 mcg/kg dexmedetomidine (Dexdomitor, Orion Pharma, Espoo, Finland) and 1 mg/kg ketamine (Ketavet 100, Intervet Productions S.r.l., Aprilia, Italy)]; in cats [(0.2 mg/kg methadone (Semfortan, Dechra Pharmaceuticals PLC, Northwich, UK), 10 mcg/kg dexmedetomidine (Dexdomitor, Orion Pharma, Espoo, Finland) and 2 mg/kg ketamine (Ketavet 100, Intervet Productions S.r.l., Aprilia, Italy)]. The interventional procedures were performed with the animal left on spontaneous ventilation and kept under sedation with propofol (Propovet, Zeoetis, Parsippany, NJ, USA).

The animals were monitored by the anesthetist throughout the procedure and vital signs were recorded every five minutes on a special medical record. The patients’ recovery was then monitored by the anesthetist until the animals woke up and had fully returned to normal. Similarly, for CT-guided biopsies, which were all performed at the Pet Care Clinic in Bologna, two different premedication protocols were used. In dogs [0.2 mg/kg methadone (Semfortan, Dechra Pharmaceuticals PLC, Northwich, UK), 5 mcg/kg dexmedetomidine (Dexdomitor, Orion Pharma, Espoo, Finland) and 1 mg/kg ketamine (Ketavet 100, Intervet Productions S.r.l., Aprilia, Italy)]; in cats [(0.2 mg/kg methadone (Semfortan, Dechra Pharmaceuticals PLC, Northwich, UK), 10 mcg/kg dexmedetomidine (Dexdomitor, Orion Pharma, Espoo, Finland) and 2 mg/kg ketamine (Ketavet 100, Intervet Productions S.r.l., Aprilia, Italy)]. Unlike US-guided biopsies, CT-guided interventional procedures were performed with the animal intubated, under 2% isoflurane gas anesthesia (Vetflurane, Virbac, Carros, France) and intraoperative fluid therapy (Ringer lactate, S.A.L.F. S.p.A., Bergamo, Italy), rate 10 mL/kg/h). During the procedure, all the animal’s vital signs were monitored by the anesthetist. At the end of the imaging-guided biopsy, the animals were given atipamezole (Atipam, Dechra Veterinary Products S.r.l, Torino, Italy) (30 mcg/kg for dogs and 100 mcg/kg for cats) to wake them up.

The US/CT scan of the masses and the collection of tissue samples for extemporaneous cytology and histology were performed after trichotomy and disinfection of the skin at the site of the tru-cut/bone needle insertion. Dogs and cats that met the following inclusion criteria were enrolled in the study: patients with at least one soft tissue and/or bone masses; patients in good health, with a normal cardiovascular examination, hematobiochemical tests and coagulation profile with normal platelet count. All animal owners were informed of the interventional procedure and the related anesthetic risks. For each procedure performed, the owner was asked to sign an informed consent form.

### 2.1. Image-Guided Biopsies

**Figure 1:**
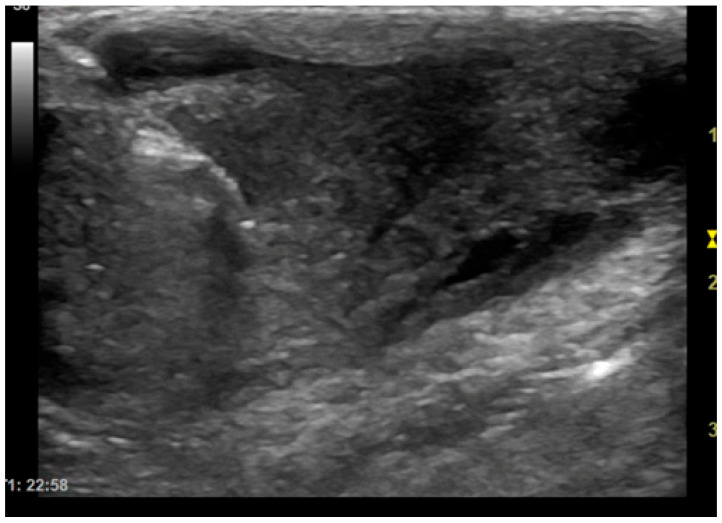
Dog, *Mixed breed*, 5 y, neutered male. Percutaneous ultrasound-guided biopsy of a subcutaneous mass. Final diagnosis: pyogranuloma.

**Figure 2:**
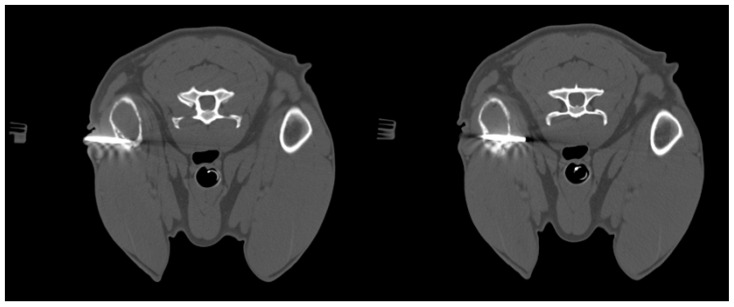
Dog, *Mixed breed*, 13 y, spayed female. Phases of CT-guided humeral biopsy with bone window. Final diagnosis: humeral osteosarcoma.

The patients included in the study underwent imaging-guided biopsies, specifically under US or CT guidance, of the masses under examination as shown in Figure 1 and Figure 2. Biopsy samples were collected using a semi-automatic spring-loaded tru-cut (14 G) in the case of soft tissue lesions and a bone needle (8–10 G) for bone tissue. These interventional diagnostic methods were performed after monitoring and anesthetizing the patient, under the continuous supervision of the anesthetist in charge. Trichotomy of the area of interest was performed, followed by surgical scrubbing with betadine and alcohol. Once the patient was prepared, a small incision was made with a number 11 scalpel blade on the skin surface, through which the tru-cut (14 G) or bone needle (8–10 G) was subsequently inserted. The biopsies were performed using ultrasound guidance or computed tomography, depending on the case, the location of the lesion and the availability of instruments.

### 2.2. Extemporaneous Cytology

While the patient was being prepared, a second operator arranged the cytology workspace. All the slides were laid out on a large surface and labeled with the animal’s details, the date of collection, the location and the type of lesion. Once the biopsy sample had been collected, the second operator used a hypodermic needle to transfer the tissue just removed from the tru-cut or bone needle onto the slide. The tissue core was then gently rolled onto the slide, creating two parallel lines, rolling first in one direction and then in the opposite direction, to obtain a good single cell layer for reading under an optical microscope. Approximately three slides were prepared for each biopsy sample, which were then immediately air-dried.

After drying, slides were sent to the cytology laboratory of the facility where the interventional procedure had been performed and stained using manual or automatic (Aerospray^®^ Hematology Slide Stainer/Cytocentrifuge 7120, Wescor, Inc., Logan, UT, USA) May-Grünwald-Giemsa method. The slides were examined under an optical microscope 60X Plan Apo oil-immersion objective (Eclipse^®^ E600, Nikon, Tokyo, Japan) immediately after the biopsy material was collected. In this way, within 10–15 min, information was obtained on the presence or absence of cellular material representative of the sampled lesion. The duration of cytologic assessment did not affect the patient’s stability during the procedure. When representative cytologic material was obtained, the procedure was concluded, and the patient was monitored until full recovery. If the samples showed low cellularity or necrotic material, the biopsy was repeated whenever feasible. The specimens were considered cytologically adequate if they contained a sufficient number of well-preserved and representative cells, properly distributed and free from significant artifacts, such us air-drying or crush artifacts, obscuring blood or inflammation, thick or uneven smears, and poor fixation or staining artifacts.

### 2.3. Histopathology

Finally, all biopsy samples were preserved for histological examination. The tissue cores, after being rolled using sterile hypodermic needles onto appropriately labeled slides, were stored in special containers, immersed in 10% formalin and appropriately labeled. All samples were subjected to histopathological examinations.

### 2.4. Statistical Analysis

The accuracy of immediate cytology from imaging-guided biopsies performed with tru-cut or bone needles was evaluated by comparing the results obtained with histological diagnoses (gold standard), both to assess the cellularity adequately representative of the sampled lesion and to evaluate the diagnostic capacity of cytology itself. The data were analyzed using statistical software (MedCalc 17.9.2, MedCalc Software, Gand, Belgium), and a significance level of *p* < 0.05 was set for all statistical analyses.

## 3. Results

The study enrolled 79 animals, including 71 (89.9%) dogs and 8 (10.1%) cats of different breeds, sizes and sexes that had at least one mass affecting soft tissue and/or bone tissue. In the group of 71 dogs, 36 (50.7%) were female (of which 30 were spayed and 6 were intact) and 35 (49.3%) were male (of which 19 were neutered and 16 were intact). In the group of 8 cats, 3 (37.5%) were spayed females and 5 (62.5%) were intact males.

Among dogs, the average age was 9.1 years (±3.8 standard deviation—SD), while among cats it was 9.9 years (±4.2 SD). Biopsies were performed under ultrasound guidance (US) in 57/79 (72.2%) cases, and the remaining 22/79 (27.8%) were performed under computed tomography (CT) guidance.

Table 1 summarizes all the data collected (histological diagnosis, cytological diagnosis, mass location and cellularity of the extemporaneous cytology).

In this study, histological reports from imaging-guided biopsies were considered the gold standard. Regarding the extemporaneous cytological examination of representative cells from the biopsied lesion, 64/79 samples were found to be cellular and representative.

Among the 15 cytological samples lacking representative cellularity: 7 were poorly stained and/or poorly fixed; 7 samples were contaminated with blood; and 1 sample lacked cellularity. Therefore, the accuracy and sensitivity for the presence of cells representative of the sampled tissue were 81.1% (95% confidence interval [CI], 72.5–89.7).

As regards cytological diagnosis, it was possible and correct in 53/79 cases. Of these, 37 were neoplastic masses, 10 were inflammatory lesions, 4 were hyperplasia, one of each for necrosis, vacuolar hepatopathy, and follicular cyst. Among the neoplastic masses, lipomas, sarcomas, and carcinomas were the most frequently identified types. Furthermore, of the 26 cases with a mismatch between cytological and histological diagnosis (reported in Table 2), 21 were non-diagnostic due to poor cellularity or because they were blood-contaminated or poorly stained/fixed, and 5 were due to a diagnosis that did not correspond to the histopathology.

Therefore, the overall accuracy for the correspondence between cytological and histological diagnoses was 68.3% (95% CI, 58.0–78.5).

Excluding cytological samples that were non-diagnostic due to incorrect sample preparation or the intrinsic nature of the lesions sampled, the cytological and histological diagnoses did not match in 5/58 cases, with an overall accuracy of 92.1% (95% CI, 85.1–99.0) for the extemporaneous cytology.

## 4. Discussion

In this study, US or CT-guided biopsies were performed using a semi-automatic spring-loaded tru-cut (in the case of soft tissue lesions) and bone needle (in the case of bone lesions). In veterinary medicine, as in human medicine, diagnostic imaging is an essential step in the diagnostic process for many bone and soft tissue lesions [2]. However, imaging techniques (radiology, ultrasonography, computed tomography, fluoroscopy, magnetic resonance imaging) are often unable to identify the exact nature of a lesion, for example, distinguishing between inflammatory/infectious diseases and neoplastic diseases. Therefore, biopsy is essential to characterize a lesion with certainty [1].

To the author’s knowledge, there is no literature in veterinary medicine concerning the procedure described in this paper. The reason for conducting this study lies precisely in the fact that biopsy samples may be non-diagnostic, which, in addition to causing understandable frustration for the doctor and owner, implies a missed diagnosis, prolonged delays in obtaining a definitive diagnosis and initiating the appropriate treatment protocol, the need to reschedule the interventional procedure, additional costs for the owner, and an increased risk of complications for the animal, which is forced to undergo anesthesia and an invasive procedure again.

The rapid on-site evaluation (ROSE) technique, first proposed in 1981, was designed to provide immediate feedback on sample adequacy during the procedure, to guide the operator in modifying the sampling technique (e.g., site or depth of sampling), and to allow for a rapid preliminary diagnosis [10].

As demonstrated in some studies in human medicine, the diagnostic accuracy of imaging-guided biopsy samples, performed without prior extemporaneous cytological evaluation, is lower [10]. In fact, the diagnostic accuracy of lung lesion biopsies without prior cytological evaluation was assessed, with accuracy values ranging from 65% to 94% [11,12]. On the other hand, the use of ROSE led to a 12% improvement in sampling adequacy and a 13% increase in diagnostic accuracy compared to groups without ROSE.

No significant differences in complication rates were found between groups with and without ROSE. The combination of imaging-guided biopsy and ROSE has been shown to improve diagnostic accuracy without extending the procedure time or increasing the risk of complications.

**Figure 3:**
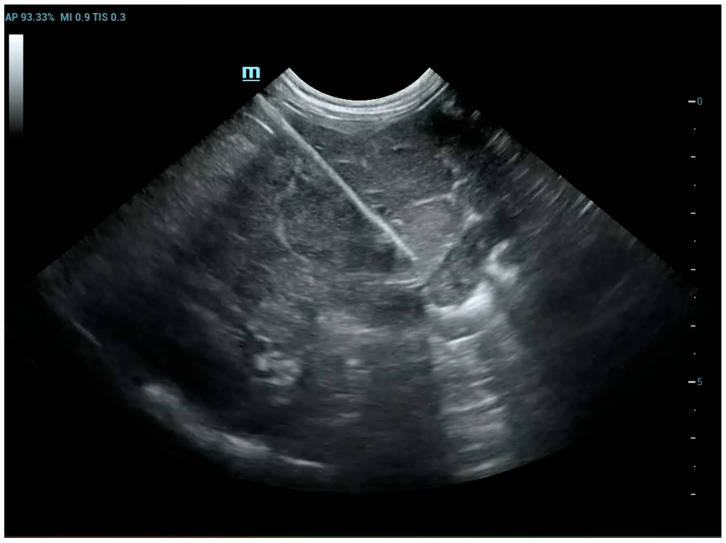
Dog, *Mixed breed*, 8 y, spayed female. Percutaneous ultrasound-guided biopsy of liver mass. Final diagnosis: Well-differentiated carcinoma.

ROSE provides immediate feedback on sample adequacy, enabling a faster and more reliable diagnosis [10]. To increase the likelihood of obtaining diagnostic samples, multiple biopsies are usually performed on the same lesion. However, performing each biopsy, especially in dangerous anatomical locations, can cause complications. Therefore, the main objective of this study is to try to reduce the number of biopsies that need to be performed to obtain a diagnostic sample, thereby also lowering the risks for the animal. Analysis of the results of this study has shown that extemporaneous cytological examination can be useful for the purpose described above. In fact, out of 79 biopsies collected, extemporaneous cytological examination revealed cellularity in 81% of cases. The presence of representative cellularity in these samples made it possible to terminate the biopsy procedures. In the remaining cases, the lack of cellularity in the extemporaneous cytology suggested that further sampling should be carried out. Furthermore, it should be noted that in most of the samples in which no cellularity representative of the biopsied lesion was detected (25 out of 79), the cause was attributed to poorly stained and/or poorly fixed slides (technical problems), the presence of blood-contamination and poor cellularity due to the intrinsic nature of the sampled lesion. During the oncogenic process, blood may not arrive in sufficient quantities, causing an environment that is poor in oxygen (hypoxic) and nutrients (ischemic), which leads to necrosis, thus reducing the cellularity of the sample [13,14]. Furthermore, it is important to note that the interventional procedures in this study, excluding those performed with CT, were performed with 2D-mode ultrasound as shown in Figure 3. CT-guided biopsy sampling is, in fact, a procedure mainly indicated for bone lesions or lesions located in areas not easily accessible with US [6,15]. The relevance of this has been demonstrated in several studies reported in the literature as it can lead to several advantages. In the study by Vignoli et al. [16], in 2013, the importance of CT and guided biopsy in the staging of cancer patients was emphasized. In fact, although rare, muscular metastases can occur with different types of neoplasms. The most common sites of metastasis included the cervical, thoracic and lumbar paraspinal muscles, the chest wall, the scapular region, the hind limbs and, more rarely, the abdominal wall. The results highlight the importance of CT in the staging of cancer in dogs and cats, particularly in tumors with a high propensity for metastasis [14]. In this regard, for example, another study highlighted the frequency of muscle metastases in cases of hemangiosarcoma. Muscle metastases were found in 15/60 dogs (24.6%), all of which also had metastases in other organs. These data suggest that the use of whole-body CT and guided biopsy is essential in the staging of dogs with hemangiosarcoma, as muscle metastases may not be detected by clinical examination alone or by traditional imaging methods such as radiography and ultrasound [17]. Moreover, the intravenous injection of a non-ionic iodinated contrast medium provides additional vascular information about the lesion, allowing biopsy of viable tissue while avoiding major blood vessels and to sample the most representative portions of the lesion [8]. On the other hand, ultrasound is the method of choice in daily practice due to the wide availability of US equipment and its lower cost compared to CT [18]. In fact, as reported in some human literature, sampling of lung lesions visible with US is preferable [19]. Ultrasound can be used for real-time, multiplanar visualization, as well as for the accurate localization of target lesions that move with respiration. It also allows precise needle adjustment throughout all procedural steps. Additional advantages of ultrasound include the absence of radiation-related risks, its safety, speed, and cost-effectiveness [20]. Therefore, ultrasound has been recommended as an excellent option for guiding biopsies of peripheral pulmonary lesions [19]. Moreover, as demonstrated in some studies, the injection of contrast-enhanced ultrasound (CEUS) allows for the precise definition of the margins of the lesion, the identification of any necrotic areas, and the assessment of the distribution of vessels within it. The use of CEUS may be recommended to directly guide sampling procedures, improving the diagnostic success of cytological and histological examinations [21,22]. Therefore, paying particular attention to avoiding pre-analytical errors in the collection and/or preparation of slides, this method could prove particularly useful in interventional diagnostics.

Biopsy complications can be broadly divided into minor and major complications. Minor complications, most limited to hemorrhage or mild pneumothorax in the case of lung biopsy, occur in 5.6–21.9% of cases. Major complications requiring intervention (e.g., fluid therapy, blood transfusion, chest drainage, surgery) are reported in 1.2–6% of cases [23]. Significant hemorrhage has been observed in thrombocytopenic dogs, those with prolonged One-Stage Prothrombin Time (OSPT) and cats with prolonged activated Partial Thromboplastin Time (aPTT). Moreover, the complication rate was organ-dependent (higher for the kidneys than for the liver) and all major complications occurred within ten hours of the biopsy. When there is a known potential risk, such as too low platelet count (<80,000 mcl) or too high OSPT and aPTT, biopsy should be avoided [23]. Bleeding may also occur due to unintentional injury, patient movement or poor operator technique. If the procedure is performed correctly, the risk of infection and tumor dissemination are rare events in veterinary medicine, with the exception of transitional cell carcinoma of the urogenital tract [24,25] or pulmonary carcinoma [26]. Our study was not limited to analyzing the results of the extemporaneous cytology to determine whether it was useful for identifying a representative cellularity of the biopsied sample. In fact, by comparing cytological and histological diagnoses, the diagnostic accuracy of extemporaneous cytology was evaluated, understood as the test’s ability to make the correct diagnosis, taking histological diagnosis as the gold standard. This comparison shows that cytological diagnosis, although less accurate than histology, is still quite accurate, with the advantage that it is immediate and there is no need to wait for the histopathology results.

In particular, the diagnostic accuracy calculated in this study for the cytological technique of rolling cores from imaging-guided biopsies was 68%, a value that could be improved with greater attention to slide preparation. In fact, of the 25 samples with a mismatch between cytological and histological diagnoses, 21 were found to be non-diagnostic. In particular, in 7 cases, poor fixation and/or staining of the slides was found, which affected their reading, while in a further 7 samples, blood-contamination was present: in two cases in particular, these were lesions affecting the vascular system (1 hemangioma, 1 hemangiosarcoma); therefore, the presence of red blood cells was compatible with the type of lesion under examination. In one case of lymphoplasmacytic inflammation, no cells were present. Furthermore, in 2 cases it was not possible to diagnose hyperplasia as quantitative cell assessment is not always possible cytologically; the gold standard method for diagnosing tissue hyperplasia is histology; in 1 case, cytology was defined as non-diagnostic, as cellularity is almost non-existent during fibrosis [27]. In two cases, sarcomatous lesions (one hemangiosarcoma and one fibrosarcoma) were not diagnosed because most sarcomas show little or no exfoliation, often as individual cells [28]. Finally, one case of well-differentiated carcinoma was not identified; the literature reports that carcinomas and round cell tumors exfoliate much better than sarcomas [1]; in this case, the fact that the sample is non-diagnostic could be justified by the difficult cytological differentiation between a well-differentiated carcinoma and normal cells [29].

It was also observed that, among the neoplastic masses included in our study, lipomas, melanomas and mast cell tumors were the lesions most frequently diagnosed correctly. For these types of lesions, in fact, the extemporaneous cytological examination performed by rolling the tissue core showed 100% diagnostic accuracy, proving to be as accurate as histopathological examination. For lipomas, a biopsy is performed when the mass has atypical features or if there is any doubt that it is a harmless lipoma. Most lipomas in dogs are benign and only need monitoring unless they grow or cause discomfort. In addition to calculating the diagnostic accuracy of this method, the results obtained in our study were also compared with data on FNA found in the literature. This comparison shows that the diagnostic accuracy of extemporaneous cytology from core samples is on average in line with that obtained from samples taken with a fine needle. With 68% diagnostic accuracy, the results of the extemporaneous cytological examination were like those previously found by Zekas and colleagues who reported a diagnostic accuracy for FNA of 65% (compared to 83% for TCB) in 30 cases of intrathoracic lesions in dogs and cats [6]. A more recent study, in 2021, conducted on 62 thoracic masses in dogs and cats, found that the diagnostic accuracy of FNA was 67.7% compared to 95.2% for TCB [7]. In another study involving 21 dogs and 2 cats with bone lesions, has been reported that FNA had a diagnostic accuracy of 83.3% compared to 100% for TCB [8]. In one study describing intrathoracic lesions, samples collected via CT-guided FNA revealed the presence of carcinoma in three out of four cases, with a diagnostic accuracy of 75% [30]. In our study, diagnostic accuracy is lower, although it should be noted that the study in question has the limitation of having considered a small number of cases [30]. In other anatomical sites, the literature reports variable concordance rates between cytologic and histologic diagnoses. For example, in the study by Wang et al. [31], the concordance between cytology and histology for hepatic lesions was 30% in dogs and 51% in cats, based on a total of 97 subjects. It should be noted that this study also included cases of diffuse hepatic disease, which may have affected diagnostic agreement. In another investigation, the concordance between cytologic and histologic diagnoses of splenic lesions was 61%; however, the sample size was limited to only 32 patients, reducing the statistical robustness of the findings [32].

In our study, on the other hand, various types of lesions were analyzed, including some types of tumors with low exfoliation (e.g., sarcomas), others such as hyperplasia, which require a quantitative assessment of cellularity that is not always possible in cytology, and others such as fibrosis, which has almost no cellularity [27]. In human medicine, several studies have investigated the diagnostic accuracy of FNA. In one retrospective study, 97 ultrasound-guided fine needle cyto-biopsies were performed in 92 patients with one or more suspicious liver lesions. The cyto-biopsy results were compared with the final diagnosis obtained from histological examination or follow-up. In 65 confirmed malignant tumors, the fine needle biopsy was concordant in 54 cases, showing a diagnostic accuracy of 83% [33]. In another recent study involving 152 patients with palpable breast lesions, all underwent Fine Needle Aspiration Cytology and Core Needle Biopsy; the results showed that both methods had similar diagnostic accuracy rates (approximately 94%) [34]. In conclusion, both objectives of this study were achieved with good results. The usefulness of extemporaneous cytological examination in image-guided biopsies was demonstrated, with high accuracy values obtained both for the presence of cellularity representative of the biopsied sample (81%) and for the cytological diagnosis compared with the histological diagnosis (68%).

This study has several limitations. First, although 79 animals were included, the study population was heterogeneous, encompassing both dogs and cats with a wide range of lesion types and anatomical locations. This variability could have influenced the diagnostic accuracy of extemporaneous cytology, as different tissue types (e.g., sarcomas vs. epithelial tumors) show markedly different exfoliation properties. A larger, more homogeneous sample could provide more precise estimates of sensitivity and accuracy for each group. Second, the study evaluates the extemporaneous cytology technique but does not include a direct comparison with other rapid on-site evaluation (ROSE) methods, which are already established in human medicine. Including a comparative arm could help determine whether this simplified veterinary adaptation offers comparable or superior performance in terms of sample adequacy and diagnostic yield. In addition to the limitations previously mentioned, it is important to note that the procedure described in this study can only be implemented in facilities where multiple specialized professionals are available to correctly and safely perform all stages of the intervention.

## 5. Conclusions

The results of this study demonstrate that immediate cytological examination can be useful for verifying the presence of cells, limiting the number of biopsies to be performed and therefore limiting the risks associated with them. This method could therefore prove useful in interventional diagnostics, as in the presence of cytologically representative samples from the biopsy procedure, it would allow us to terminate the biopsy procedures. This practical implication is particularly important for biopsies in dangerous locations, as it would lower the risk of complications (e.g., intra-abdominal hemorrhage, biliary peritonitis, liver laceration, pneumothorax, translocation of neoplastic cells, e.g., in transitional cell carcinoma of the urogenital tract, etc.). Although less precise than histology, cytological diagnosis still has good diagnostic accuracy, with the advantage that it is immediate and there is no need to wait for the histopathology results. Specifically, it has been shown to be particularly accurate for the diagnosis of mast cell tumors, lipomas and melanomas. Furthermore, it has shown a diagnostic accuracy like that reported in the literature on cytological examination of samples collected by fine needle aspiration. Finally, it has been seen that paying greater attention to the preparation of slides and sampling methods could improve both the accuracy of the search for cellularity representative of the sampled lesion and the diagnostic accuracy of this method. Obviously, it should be noted that histology remains the gold standard method for reaching a definitive diagnosis of neoplastic lesions affecting bones or soft tissues. Cytology and histopathology therefore remain two complementary diagnostic procedures, reflecting a compromise between the lower degree of invasiveness of cytology sampling and the greater amount of information available thanks to histopathology.

## Figures and Tables

**Table 1:** Histological and cytological diagnosis, location of the lesion and type of cellularity found in the slides analyzed extemporaneously at biopsy, rolling the tissue core.

Histological Diagnosis (*n*)	Cytological Diagnosis (*n*)	Mass Location	Immediate Roll Cytology Cellularity
Lipoma (7)	Lipoma (7)	Various	Adequate
Melanoma (4)	Melanoma (4)	Various	Adequate
Mast cell tumor (4)	Mast cell tumor (4)	Various	Adequate
Osteosarcoma (5)	Osteosarcoma (5)	Various	Adequate
Liposarcoma (1)	Liposarcoma (1)	Subcutaneous	Adequate
Hemangiosarcoma (2)	Non-diagnostic (1)	Kidney	Low
	Non-diagnostic (1)	Subcutaneous	Poor staining/fixation
Fibrosarcoma (2)	Non-diagnostic (1)	Mouth	Low
	Non-diagnostic (1)	Subcutaneous	Poor staining/fixation
Sarcoma (6)	Sarcoma (5)	Various	Adequate/moderate
	Non-diagnostic (1)	Cervical muscles	Blood contamination
Lymphoma (4)	Lymphoma (2)	Lymph node	Adequate
	Lymphoma (1)	Mediastinum	Adequate
	Non-diagnostic (1)	Liver	Poor staining/fixation
Carcinoma (12)	Carcinoma (4)	Lung	Adequate
	Carcinoma (2)	Anal sacs	Adequate
	Carcinoma (1)	Nose	Adequate
	Carcinoma (1)	Prostate	Adequate
	Non-diagnostic (2)	Liver	Low-Poor staining/fixation
	Non-diagnostic (2)	Subcutaneous	Poor staining/fixation- Blood contamination
Purulent inflammation (4)	Purulent inflammation (4)	Various	Adequate
Pyogranuloma (4)	Pyogranuloma (4)	Various	Adequate
Lymphoplasmacytic inflammation (4)	Lymphoplasmacytic inflammation (1)	Stomach	Adequate
	Non-diagnostic (1)	Maxillary region	Blood contamination
	Non-diagnostic (1)	Temporal muscle	Acellular
	Non-diagnostic (1)	Liver	Poor staining/fixation
Mixed inflammation (4)	Mixed inflammation (1)	Subcutaneous	Adequate
	Osteosarcoma (1)	Radius	Low
	Non-diagnostic (1)	Thoracic wall	Blood contamination
	Non-diagnostic (1)	Anal sacs	Blood contamination
Hyperplasia (5)	Hyperplasia (3)	Prostate	Adequate
	Non-diagnostic (2)	Liver	Low-Moderate
Hypoperfusion (2)	Non-diagnostic (1)	Liver	Poor staining/fixation
	Normal (1)	Liver	Moderate
Fibrosis (2)	Non-diagnostic (1)	Kidney	Low
	Non-diagnostic (1)	Mammary gland	Blood contamination
Necrosis (2)	Necrosis (1)	Maxillary region	Low
	Dysplasia (1)	Kidney	Low
Vacuolar hepatopathy (1)	Vacuolar hepatopathy (1)	Liver	Adequate
Tubular epithelial degeneration (1)	Normal (1)	Kidney	Adequate
Hamartoma (1)	Eosinophilic inflammation (1)	Nose	Low
Hemangioma (1)	Non-diagnostic (1)	Subcutaneous	Blood contamination
Follicular cysts (1)	Follicular cysts (1)	Subcutaneous	Adequate

**Table 2:** Cases with discrepancies between cytological and histological diagnosis (26/79 = 32.9%).

Histological Diagnosis (*n*)	Number of Cases
Hemangiosarcoma (2)	2
Fibrosarcoma (2)	2
Sarcoma (6)	1
Lymphoma (4)	1
Carcinoma (12)	4
Lymphoplasmacytic inflammation (4)	3
Mixed inflammation (4)	3
Hyperplasia (5)	2
Hypoperfusion (2)	2
Fibrosis (2)	2
Necrosis (2)	1
Tubular epithelial degeneration (1)	1
Hamartoma (1)	1
Hemangioma (1)	1

## Data Availability

The original contributions presented in this study are included in the article. Further inquiries can be directed to the corresponding author.

## Abbreviations

The following abbreviations are used in this manuscript:

NM	Neutered male
Y	Years
F	Female
M	Male
SF	Spayed female
US	Ultrasound
CT	Computed tomography
MRI	Magnetic resonance
FNA	Fine needle aspiration
FNB	Fine needle biopsy
TCB	Tru-cut biopsy
G	Gauge
aPTT	Activated Partial Thromboplastin Time
OSPT	One-Stage Prothrombin Time
